# Fetal goiter identified in a pregnant woman with triiodothyronine-predominant graves’ disease: a case report

**DOI:** 10.1186/s12884-020-03035-2

**Published:** 2020-06-03

**Authors:** Akiko Fujishima, Akira Sato, Hiroshi Miura, Yuki Shimoda, Saeko Kameyama, Chika Ariake, Hiroyuki Adachi, Yuki Fukuoka, Yukihiro Terada

**Affiliations:** 1 Department of Obstetrics and Gynecology, Akita University Hospital, 1-1-1 Hondo, Akita, 010-8543 Japan; 2 Perinatal Medical Center, Japanese Red Cross Akita Hospital, Akita, Japan; 3 Department of Neonatal Medicine, Akita University Hospital, Akita, Japan; 4 Department of Diabetes and Endocrinology, Akita University Hospital, Akita, Japan

**Keywords:** Triiodothyronine-predominant graves’ disease, Fetal goiter, Perinatal management, Antithyroid drug, Block-replace therapy, Case report

## Abstract

**Background:**

Approximately 10% of all Graves’ disease cases are triiodothyronine (T3)-predominant. T3-predominance is characterized by higher T3 levels than thyroxine (T4) levels. Thyroid stimulating hormone receptor autoantibody (TRAb) levels are higher in T3-predominant Graves’ disease cases than in non-T3-predominant Graves’ disease cases. Treatment with oral drugs is difficult. Here, we report a case of fetal goiter in a pregnant woman with T3-predominant Graves’ disease.

**Case presentation:**

A 31-year-old woman had unstable thyroid function during the third trimester of pregnancy, making it impossible to reduce her dosage of antithyroid medication. She was admitted to our hospital at 34 weeks of gestation owing to hydramnios and signs of threatened premature labor, and fetal goiter (thyromegaly) was detected. The dose of her antithyroid medication was reduced, based on the assumption that it had migrated to the fetus. Subsequently, the fetal goiter decreased in size, and the hydramnios improved. The patient underwent elective cesarean delivery at 36 weeks and 5 days of gestation. The infant presented with temporary symptoms of hyperthyroidism that improved over time.

**Conclusions:**

The recommended perinatal management of Graves’ disease is to adjust free T4 within a range from the upper limit of normal to a slightly elevated level in order to maintain the thyroid function of the fetus. However, in T3-predominant cases, free T4 levels may drop during the long-term course of the pregnancy owing to attempts to control the mother’s symptoms of thyrotoxicosis. Little is known about the perinatal management and appropriate therapeutic strategy for T3-predominant cases and fetal goiter. Therefore, further investigation is necessary.

## Background

Fetal goiter is a diffuse enlargement of the fetal thyroid gland that can occur regardless of the status of the thyroid’s function. However, it has been reported that a fetal goiter is often associated with a change in thyroid function [1]. Fetal goiter is an extremely rare condition that affects one out of 30,000–50,000 pregnancies [2]. In pregnant women with Graves’ disease, the antithyroid drug or thyroid stimulating hormone (TSH) receptor autoantibody (TRAb) migrates across the placenta, increasing the fetus’ risk of goiter and thyroid dysfunction. This dysfunction can carry over into the neonatal period. Antithyroid drugs administered during pregnancy have a stronger suppressive effect on the fetus’ thyroid function than on the mother’s thyroid function. As a result, both the Graves’ Disease Guidelines (Japan Thyroid Association) [3] and the Guidelines of the American Thyroid Association [4] recommend that the mother’s free thyroxine (FT4) levels be adjusted to a range within the upper limit of normal to slightly elevated compared to those in a non-pregnant woman. However, in the case of triiodothyronine (T3)-predominant Graves’ disease, which accounts for approximately 10% of all Graves’ disease cases, if the FT4 levels are controlled as described above, then the free triiodothyronine (FT3) levels become elevated, thus preventing suppression of the mother’s hyperthyroidism symptoms. It is difficult to achieve remission with oral medication in women with T3-predominant Graves’ disease; the recommended treatment is either isotope therapy or surgery [5].

In suspected cases of fetal thyroid dysfunction, the antithyroid drug dose administered to the mother is increased when the results of percutaneous umbilical blood sampling (PUBS) and/or fetal ultrasound indicate fetal hyperthyroidism. However, for fetal hypothyroidism, it is useful to decrease the mother’s antithyroid dose, and administer levothyroxine in the amniotic fluid [6, 7].

Here, we report the case of a pregnant woman with T3-predominant Graves’ disease that was challenging to manage and resulted in fetal goiter.

## Case presentation

A 31-year-old woman (G1, P0) had been diagnosed with Graves’ disease at 16 years of age. She discontinued treatment at her own discretion but was advised to resume treatment when she expressed her desire to become pregnant. At the time treatment had resumed, her TRAb (second generation) level was 211 IU/L (normal range: < 1.75 IU/L). Her family history was negative.

### History of current disease

The patient became pregnant naturally, at a time when her thyroid function was not satisfactorily controlled by a high dose of propylthiouracil (PTU). An alternative treatment (isotope therapy or surgery) was being considered. Because of hyperemesis gravidarum, oral medications were no longer an option, resulting in her thyroid function deteriorating during week 13 of her pregnancy. Her TRAb level was 43.6 IU/L at that time, and a daily dose of 50 mg potassium iodide (KI) was administered. As a result, her thyroid function was under control, and KI administration was discontinued at week 17. The patient’s FT3 level fluctuated between normal and elevated, whereas her FT4 and TSH levels were low. Her TRAb level gradually declined but was still high at week 33 (18.4 IU/L).

At 33 weeks and 5 days of gestation, the amniotic pocket and the cervical lengths were 8.1 and 2.6 cm, respectively, indicating worsening hydramnios and threatened premature labor. A blood test showed that the patient’s TSH level had increased to within the normal range. Because pre-pregnancy reduction in her antithyroid medication caused sudden worsening of her thyroid function, she was prescribed a daily supplementary course of 25 μg levothyroxine. At 34 weeks of gestation, she was admitted for hydramnios management and prevention of threatened premature labor.

### Progress of post-admission therapy

**Fig. 1:**
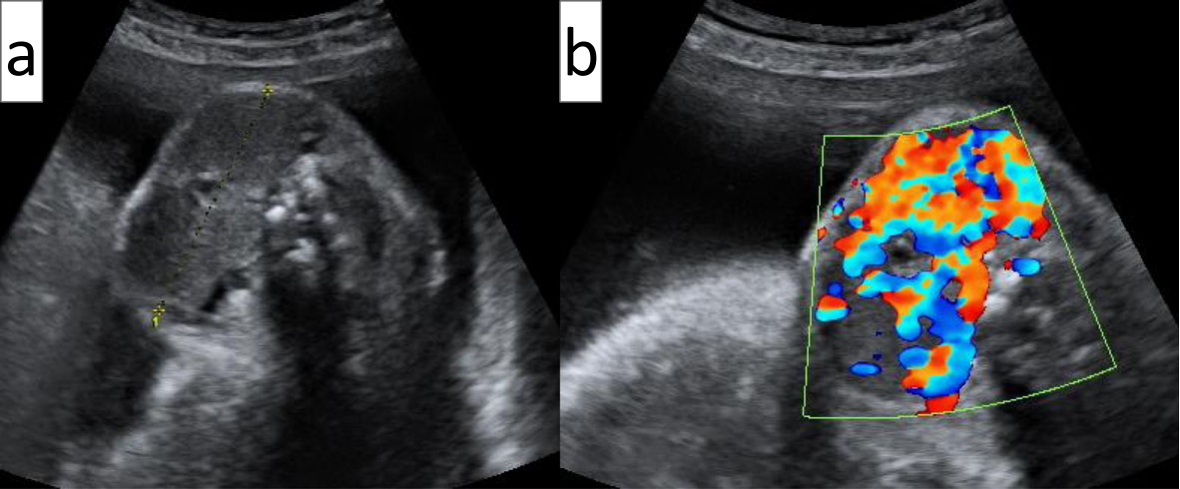
Fetal ultrasound images. Both images are taken at 34 weeks and 4 days of gestation. **a**: An image of the transverse section of the fetal neck. The transverse diameter and circumference of the fetal thyroid were 58.4 and 178 mm, respectively. **b**: An image of the transverse section of the fetal neck obtained with an ultrasonic Doppler blood flowmeter. The blood flow was diffusely increased

**Fig. 2:**
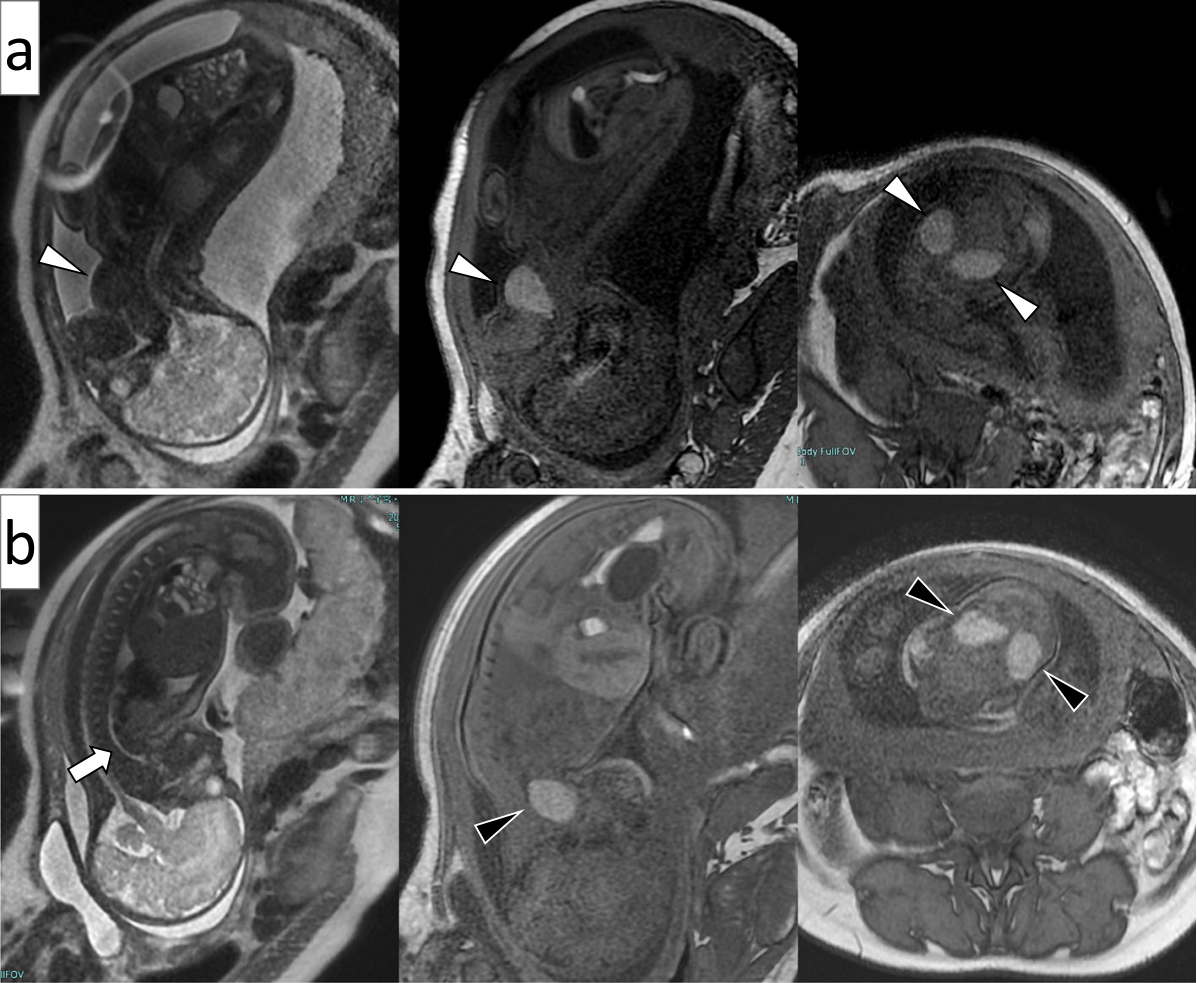
Fetal MRI images. Left: T2-weighted sagittal section of the fetus, Middle: T1-weighted sagittal section of the fetus, Right: T1-weighted transverse section of the fetus. **a**: Images taken at 34 weeks and 5 days of gestation. The anterior side of the cervical region of the fetus is swollen and protruding, and the fetus is in face presentation. The cervical region of the fetus shows a mass with left-right symmetry. T1-weighted images are hyperechoic and T2-weighted images are hypoechoic (△). **b**: Images taken at 36 weeks and 3 days of gestation. The cervical mass of the fetus (▲) shows no change in the MRI intensity, although it does show slight tendency toward shrinkage. The fetus is in a normal, flexed presentation, and the volume of amniotic fluid has decreased. A T2-weighted sagittal section of the fetus confirms the fetal airway (⇧)

On admission, blood sampling showed an increased TSH level; thus, her thyroid function had improved. Because she was experiencing frequent uterine contractions, we consulted the Department of Diabetes and Endocrinology at our hospital and initiated a continuous drip of ritodrine hydrochloride under careful monitoring for aggravation of hyperthyroidism. At 34 weeks and 4 days of gestation, a transabdominal ultrasound indicated enlargement of the fetal thyroid. It showed a transverse diameter and circumference of 58.4 and 178 mm, respectively (mean values at the 35th week of pregnancy: 18.9 mm and 51.1 mm respectively [8]) (Fig. 1a). The blood flow was diffusely increased (Fig. 1b), and hydramnios was observed. In addition, the epiphyseal nucleus of the distal femur could not be confirmed. The fetus was in face presentation (Fig. 2a). We assumed that the cause of the hydramnios was the inability to swallow the amniotic fluid due to the size of the thyroid. We suspected that the PTU had crossed the placenta and migrated to the fetus, causing fetal goiter; therefore, we initiated a reduced dose of antithyroid medication for the mother at 34 weeks and 5 days of gestation. After reducing the PTU dose from 200 to 100 mg/day, neither the mother nor the fetus showed signs of hyperthyroidism. The dose of PTU was gradually reduced to 50 mg/day, while the levothyroxine dose was increased to 100 μg/day. The fetal thyroid showed a shrinking tendency, as magnetic resonance imaging (MRI) performed at 36 weeks and 3 days of gestation showed a 3-cm fetal thyroid transverse diameter (transverse section; Fig. 2b). The next day, an ultrasound indicated that the AFI had decreased from 24 to 15 cm, and that the fetus’ thyroid blood flow had returned to normal (restricted to the thyroid margins). The fetus had moved into a normal flexion position.

Because of the goiter, we were unable to confirm that there would be a patent airway after delivery. Therefore, we speculated that a tracheotomy would be necessary. After consultation with both the Department of Neonatal Medicine and the Department of Pediatric Surgery, an elective cesarean delivery was performed at 36 weeks and 5 days of gestation. On day 7 after delivery, the mother showed signs of hyperthyroidism, which necessitated an increase in her PTU dose.

**Fig. 3:**
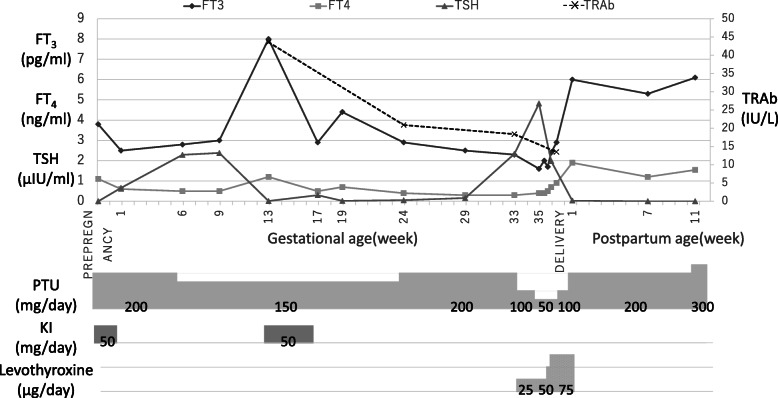
Maternal thyroid function and therapeutic course. During pregnancy, the maternal thyroid function was unstable; therefore, it was difficult to decrease the dosage of the antithyroid medication. Between the first and second trimesters, FT3 levels were normal to high, FT4 levels were low, and TSH levels were low. Elevated TSH levels were detected starting from week 33, along with a fetal goiter. Thus, the antithyroid medication dose was reduced and maintained until delivery. After delivery, hyperthyroidism was diagnosed, and the antithyroid medication dose was increased

A diagram describing the therapeutic course from pre-pregnancy to postpartum is shown in Fig. 3.

### Neonatal progress

**Fig. 4:**
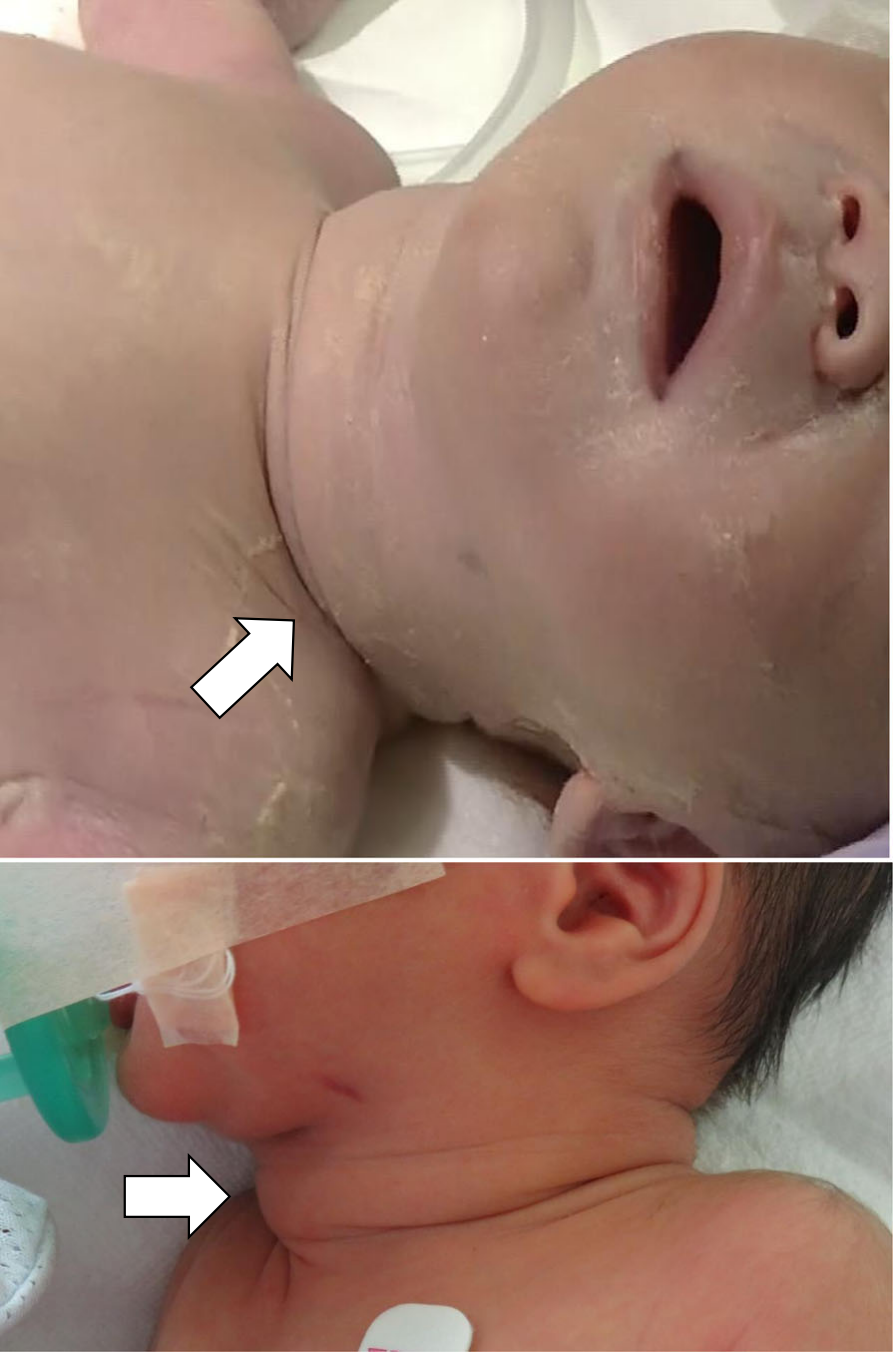
Photographs of the neonate’s neck region. The anterior cervical region has a transverse diameter of 40 mm, indicating thyroid gland enlargement

The infant was a male, weighing 2817 g, with Apgar scores of 8 and 9 at 1 and 5 min, respectively. His umbilical aortic blood pH was 7.26. The anterior cervical region had a transverse diameter of 40 mm, indicating an enlarged thyroid gland (Fig. 4). Crying was normal, and no resuscitation was necessary. The infant was admitted to the neonatal intensive care unit (NICU) immediately after birth because of premature delivery and goiter. No bradycardia was observed, and other than the goiter, no external deformities that would suggest congenital hypothyroidism, such as megaloglossia or umbilical hernia, were observed.

A sonography indicated that the thyroid gland had a left-to-right transverse diameter of 41.4 mm (normal range [mean ± 2SD]: 13.9 ± 4.0 mm [9]) and an isthmus of 5.2 mm, indicating enlargement. An ultrasound showed no increased blood flow. Radiographs indicated no epiphyseal nucleus of the distal femur, which showed that fetal bone maturation was delayed because of the fetal hypothyroidism.

Thyroid function tests were conducted using the umbilical blood at delivery, and the results were as follows: FT3, 1.7 pg/mL (normal range [mean ± SD]: 1.70 ± 0.034 pg/ml); FT4, 1.6 ng/dL (normal: 1.17 ± 0.14 ng/dl); and TSH, 25.18 μIU/mL (normal: 10.27 ± 6.31 μIU/mL) [10]. The TRAb level was 17.4 IU/L. No treatment was given until 5 days of age, when thyroid function tests indicated a tendency toward hyperthyroidism, (FT3 = 3.5 pg/mL, FT4 = 2.7 ng/dL, and TSH = 2.56 μIU/mL). The infant was administered a course of thiamazole (MMI) (0.5 mg/kg/day). No major changes in the size of the infant’s thyroid gland were observed during the hospital stay. Because of neonatal jaundice, the infant was treated with 24-h phototherapy starting at 4 days of age. His progress was stable, and he was discharged at 18 days of age, weighing 3.1 kg. Following discharge, the MMI dose was gradually decreased as TRAb levels dropped, and it was discontinued at 51 days of age. At 10 months of age, the infant’s thyroid function was normal, despite persistent goiter. There was no evidence of any developmental delays. Currently, the infant is under the continuous care of the Department of Pediatrics.

## Discussion and conclusions

In this study, we report a case of a fetal goiter in a pregnant woman with T3-predominant Graves’ disease. Pregnant women with Graves’ disease, which can cause fetal goiter, are relatively common. Graves’ disease is found in 0.1–0.4% of all pregnant women [11]. In a previous case series summarizing 11 cases involving pregnant women with Graves’ disease, two cases (19%) of fetal goiter were reported [12]. Therefore, there is a need to carefully manage such cases.

In the present case, during the 13th week of pregnancy, the patient’s TRAb level was 43.6 IU/L, indicating a high risk of fetal Graves’ disease. T3-predominant Graves’ disease is known to promote the activity of the thyroxine 5′-deiodinase (T45’D) in the thyroid gland [5], resulting in T4–to–T3 conversion within the thyroid gland. Thus, during treatment with antithyroid drugs, the T4 blood level is adjusted within the normal range, but the T3 blood level remains elevated. Remission is unsuccessful through the use of oral medications, resulting in surgery in many cases [5]. Additionally, hyperthyroidism is known to be a risk of premature delivery [13]. In the present case, because of the risk of premature birth, attempts were made to adjust the FT3 levels within the normal range. As a result, the mother’s FT4 during pregnancy was maintained at a low level. When thyroid function cannot be controlled, even with high doses of antithyroid medications, surgery is an alternative treatment. The second trimester is the optimum timing for such surgery; however, our patient expressed an aversion to surgery during pregnancy. After the 33rd week, we added levothyroxine therapy to compensate for the FT4 levels to prevent fetal hypothyroidism. However, because antithyroid medication migrates to the fetus from the mother more predominantly than T4 [14, 15], the fetus developed fetal goiter.

Either fetal hyperthyroidism or hypothyroidism can be present with a fetal goiter. A sampling of umbilical blood through PUBS is a useful technique for definitive diagnosis; however, it is known that PUBS is associated with an increased risk of fetal mortality [16]. Because the patient was already in the 35th week of pregnancy when the fetal goiter was detected, we chose not to conduct an invasive procedure, but instead opted to perform ultrasound, a minimally invasive method for assessing fetal thyroid function [17]. The ultrasound indicated increased, diffuse blood flow in the thyroid, suggesting hyperthyroidism. Nevertheless, we strongly suspected hypothyroidism on the basis of delayed development of the femur, absence of fetal tachycardia, and normal fetal movement. The use of T1-weighted MRI images in the assessment of fetal thyroid function has been reported [18]. In brief, this report assessed the thyroid-to-muscle signal intensity ratio (SIR) and showed that SIR values were significantly lower in cases of fetal hypothyroidism than in euthyroid cases. In the present case, the results suggested that thyroid function was between normal and low, and the possibility of fetal hyperthyroidism was considered low. Because we suspected fetal hypothyroidism, we reduced the dose of the antithyroid medication, which led to fetal thyroid shrinkage and a decrease in the volume of amniotic fluid.

In conclusion, fetal thyroid function in perinatal management of pregnancy with T3-predominant Graves’ disease can be tested using ultrasonography and MRI. Further research is needed to develop a standard of care for pregnant women and their fetuses when maternal Graves’ disease with T3 toxicosis is present.

## Data Availability

The datasets used and/or analyzed during the current consent study are available from the corresponding author on reasonable request.

## Abbreviations

AFI: Amniotic fluid index
CI: Confidence interval
FT3: Free triiodothyronine
FT4: Free thyroxine
IU/L: International Units/Liter
Mm: Thiamazole
MRI: Magnetic resonance imaging
NICU: Neonatal intensive care unit
pH: Potential hydrogen
PTU: Propylthiouracil
PUBS: Percutaneous umbilical blood sampling
T3: Triiodothyronine
T4: Thyroxine
T45’D: Thyroxine 5′-deiodinase
TRAb: TSH receptor autoantibody
TSH: Thyroid stimulating hormone
